# In Vitro Investigation of Statin Effects on Genes Associated with Severe COVID-19 in Cancerous and Non-Cancerous Cells

**DOI:** 10.3390/biomedicines13071714

**Published:** 2025-07-14

**Authors:** Adriana Kapustová, Patrik Macášek, Bibiána Baďurová, Jana Melegová, Silvie Rimpelová, Jan Kubovčiak, Jana Šáchová, Miluše Hradilová, Michal Kolář, Libor Vítek, Tomáš Ruml, Helena Gbelcová

**Affiliations:** 1 Institute of Medical Biology, Genetics and Clinical Genetics, Faculty of Medicine, Comenius University, Sasinkova 4, 811 08 Bratislava, Slovakia; adriana.jariabkova@fmed.uniba.sk (A.K.); patrik.macasek@fmed.uniba.sk (P.M.); badurova25@uniba.sk (B.B.); jana.melegova@uniba.sk (J.M.); 2 Department of Medical Biochemistry, Jessenius Faculty of Medicine in Martin, Comenius University, Malá Hora 4, 036 01 Marti, Slovakia; 3 Central Animal Facility, Jessenius Faculty of Medicine in Martin, Comenius University, Malá Hora 4D, 036 01 Martin, Slovakia; 4 Department of Biochemistry and Microbiology, Faculty of Food and Biochemical Technology, University of Chemistry and Technology Prague, Technická 3, 166 28 Prague, Czech Republic; silvie.rimpelova@vscht.cz (S.R.); tomas.ruml@vscht.cz (T.R.); 5 Laboratory of Genomics and Bioinformatics, Institute of Molecular Genetics, The Czech Academy of Science, Vídeňská 1083, 142 20 Prague, Czech Republic; jan.kubovciak@img.cas.cz (J.K.); jana.sachova@img.cas.cz (J.Š.); miluse.hradilova@img.cas.cz (M.H.); michal.kolar@img.cas.cz (M.K.); 6 Department of Informatics and Chemistry, University of Chemistry and Technology in Prague, Technická 3, 166 28 Prague, Czech Republic; 7 Laboratory Diagnostics, Institute of Medical Biochemistry, 4th Department of Internal Medicine, General University Hospital in Prague, 1st Faculty of Medicine, Charles University, Kateřinská 1660/32, 121 08 Prague, Czech Republic; vitek@cesnet.cz

**Keywords:** COVID-19, statins, *APOE*, *ACE2*, microarray analyses, gene expression

## Abstract

**Background:** The progressive course of coronavirus disease 2019 (COVID-19) is more frequently observed in individuals with obesity, diabetes, pulmonary and cardiovascular disease, or arterial hypertension. Many patients with these conditions are prescribed statins to treat hypercholesterolaemia. However, statins exhibit additional pleiotropic effects. The present study aims to investigate the effects of all eight currently existing statins on the expression of genes whose products have been reported to be directly associated with complicated COVID-19 disease. **Methods:** We extended the interpretation of the whole-genome DNA microarray analyses of pancreatic cancer cells MiaPaCa-2 and whole-transcriptome analyses of adipose tissue-derived mesenchymal stem cells AD-MSC that we had performed in the past. From the number of genes with altered expression induced by statins, we focused on those reported to be involved in a complicated course of COVID-19, including *APOE* and *ACE2*, genes encoding proteins involved in innate antiviral immunity and respiratory failure genes. **Results:** Although we did not observe statin-induced changes in the expression of *APOE*, *ACE2* and any of the six genes clustered in the locus associated with respiratory failure in patients with COVID-19, some statins induced changes in the expression of genes encoding their interaction partners. Among genes associated with the immune system, all statins, which are effective in vitro affected the expression of genes encoding IL-6 and IL-8 and interaction partners of NF-kB, which may influence the duration of viral persistence. **Conclusions:** Statins act on multiple pathways simultaneously, some of which support COVID-19 development, while others suppress it.

## 1. Introduction

Severe acute respiratory syndrome coronavirus 2 (SARS-CoV-2), the virus responsible for coronavirus disease 2019 (COVID-19), is a respiratory pathogen that poses significant health risks, particularly to vulnerable populations [1]. In infected individuals, the symptoms can range from a strong dry cough and fever to fatigue, and a loss of taste or smell. The severity of the disease depends on the SARS-CoV-2 variant [2,3]. Moreover, certain groups, including those over 60, individuals with diabetes, and those with pre-existing conditions such as lung and cardiovascular diseases, obesity, cancer, and hypertension, are at a higher risk of experiencing severe illness [4].

Many of these patients are prescribed statins, the 3-hydroxy-3-methylglutaryl-coenzyme A reductase (HMG-CoA reductase) inhibitors commonly used to treat high cholesterol levels. Statins work by blocking the mevalonate pathway, which is essential for synthesizing several important molecules, including isoprenoids that play vital roles in post-translational protein modifications [5]. Given their widespread use among at-risk populations, the potential influence of statins on COVID-19 progression has gained considerable attention.

Existing data on the relationship between statins and COVID-19 outcomes are rather ambiguous. Some studies have suggested that elderly patients in nursing homes who were on statin therapy experienced milder symptoms and improved clinical outcomes of COVID-19 [6]. For instance, research by Daniels et al. indicated that statin use before hospitalization was associated with a remarkable 71% reduction in the likelihood of developing severe disease. Furthermore, the use of statins was associated with a significantly faster recovery time [7]. Additionally, a retrospective analysis of nearly 14,000 COVID-19 patients revealed that those on statins had almost half the risk of 28-day all-cause mortality compared to statin-untreated patients [8]. A meta-analysis involving four studies with close to 9000 patients further supported these findings, showing a 30% reduction in severe or fatal COVID-19 outcomes among statin users compared to patients not taking statins [9]. A later meta-analysis, which examined almost 150,000 patients revealed an independent negative association of statin use and mortality among COVID-19 patients [10]. This observation was proved also in the meta-analysis of clinical studies published in 2022 and 2024 [11,12]. Conversely, some research has raised concerns about the effectiveness of statins in reducing the progression rate to severe COVID-19. A retrospective analysis in a study by Ayeh et al. found no significant association between statin use and mortality rates in COVID-19 patients and even reported an 18% increased risk of severe COVID-19 infection among statin users [13]. This contradiction may stem from the fact that upregulation of low-density lipoprotein receptors (LDL-R) incorporating cholesterol into the cell membrane via low-density lipoprotein cholesterol (LDL-C) from the plasma forms a greater number of lipid rafts which facilitates the entry of enveloped viruses, such as SARS-CoV-2, into cells, by receptor-mediated endocytosis [14]. Moreover, data from Iranian clinical studies have shown no observable benefit from statin therapy in the context of COVID-19 [15].

The contrasting results, recently reviewed by Focosi et al. [16], have left open questions regarding the overall impact of statins on SARS-CoV-2 infection and disease progression and the potential mechanisms behind any observed antiviral effects.

One of the proposed explanations for the reduced mortality associated with statin therapy are their anti-inflammatory properties, which may help to mitigate acute respiratory distress syndrome (ARDS), the leading cause of death in COVID-19 patients [17,18,19]. Statins have also been shown to have antiviral effects against other viruses, such as HIV and influenza A [20]. However, there are concerns that lowering LDL cholesterol levels may hinder the body’s natural defenses, as elevated serum concentrations of LDL cholesterol have been found to act protectively by binding and neutralizing microorganisms and their toxins [21]. Therefore, LDL cholesterol concentrations are considered to negatively correlate with increased morbidity and mortality from COVID-19 [21]. During the SARS-CoV-1 outbreak from 2002 to 2004, it was discovered that this virus enters host cells by binding to the angiotensin-converting enzyme 2 (ACE2) cell surface receptor [22]. Once attached, the SARS-CoV-1 virus employs pH-dependent endocytosis to enter the cells, using clathrin- and caveolae-independent mechanisms [22]. Additionally, cholesterol- and sphingolipid-rich lipid rafts in the cell membrane are involved in facilitating viral entry [22]. Therefore, it seems plausible that reducing or depleting cholesterol levels by statin treatment could potentially hinder the virus’s ability to enter cells by affecting the integrity of these lipid rafts. Like SARS-CoV-1, the recent SARS-CoV-2 variant responsible for the 2020 pandemic also utilizes the ACE2 receptor for cell entry [23,24,25]. However, research by Zhang et al. [8] suggests a counterintuitive effect of statins, i.e., that they may increase the risk of the SARS-CoV-2 entering cells by upregulating the *ACE2* expression.

Beyond *ACE2*, several other genes have been linked to the severity of COVID-19. Infection with SARS-CoV-1 in mice triggers a series of cellular responses through Toll-like receptors (TLRs), activating the nuclear factor kappa light chain enhancer of activated B cells (NF-κB). This activation can lead to reduced survival rates in these animals [17,26]. Experiments in mice confirmed that excessive inhibition of the TLR pathway results in severe lung damage and can ultimately lead to death due to the uncontrolled activation of compensatory signaling pathways. Inhibiting TLRs and NF-κB may lead to prolonged persistence of the virus and increase the risk of transmission between individuals [17,27,28].

Another important gene associated with COVID-19 is *APOE* encoding apolipoprotein E. This gene not only influences lipoprotein function but also plays a crucial role in the inflammatory responses of macrophages [28,29]. Patients with the *APOE* e4e4 genotype have a heightened risk of SARS-CoV-2 infection compared to those with the *APOE* e3e3 genotype, and homozygotes of *APOE* e4e4 are more likely to experience severe COVID-19 [30].

A genome-wide association study [1] conducted on DNA samples from patients in Spain and Italy identified two loci significantly associated with respiratory failure from COVID-19. One locus, 3p21.31, encompasses a cluster of six genes vital for antiviral innate immunity, including, *CCR9*, *SLC6A20*, *LZTFL1*, *CXCR6*, *XCR1*, and *FYCO1* encoding C-C chemokine receptor type 9, sodium-dependent imino acid transporter 1, leucine zipper transcription factor-like protein 1, chemokine receptor XC motif 6, chemokine XC receptor 1, and FYVE, and protein containing coiled-coil domain 1, respectively. The rs11385942-GA allele linked to decreased expression of *CXCR6* and increased expression of *SLC6A20* was found more frequently in patients requiring pulmonary ventilation. The second locus associated with COVID-19 is 9q34.2, which determines the blood type of the AB0 system. Patients with blood type A face a greater risk of lung failure compared to those with other blood types [1]. The relationship between the products of the above genes in the COVID-19 disease process and which steps of the KEGG pathway hsa05171 (Coronavirus disease—COVID-19 (Kyoto Encyclopedia of Genes and Genomes, https://www.kegg.jp, accessed 24 July 2024)) statins affect by interfering with their expression is shown in Supplementary Figure S1.

Considering the above, our in vitro study investigates the effects of statins on the expression of genes whose products have been reported to be directly associated with complicated COVID-19 disease, including *APOE*, *ACE2*, genes encoding proteins involved in innate antiviral immunity, and respiratory failure genes or their interaction partners. The elucidation of the role of statins in the reduction in disease severity and mortality at the molecular level may make them a valuable adjunctive therapy in the treatment of COVID-19.

## 2. Materials and Methods

### 2.1. Whole-Transcriptome Analyses

We extended the interpretation of the whole-genome and whole-transcriptome analyses we had performed in the past before the COVID-19 era on human cells derived from cells from the pancreatic adenocarcinoma MiaPaCa-2 (CRL-1420, ATCC) and human mesenchymal stem cells AD-MSC (PCS-500-011, ATCC), respectively [31,32]. Although the selection of experimental models was not targeted, COVID-19 and pancreatic tumorigenesis overlap in several molecular mechanisms [33,34,35]. In addition, therapies using adipose-derived mesenchymal stem cells have been reported to be responsible for the substantial clinical improvement in ICU patients [36]. The expression of selected genes in our experimental models was verified using the GeneCards database (https://www.genecards.org/, accessed on 24 July 2024).

The methods used to study the effects of statins on the gene expression profile of MiaPaCa-2 and AD-MSC cell lines were reported by Gbelcová et al. in 2017 [31] and 2024 [32], respectively.

Briefly, pure forms (≥98%) of all existing statins were used: atorvastatin, lovastatin, simvastatin, fluvastatin, cerivastatin, pravastatin, rosuvastatin, and pitavastatin (LKT Laboratories, St. Paul, MN, USA). Statins were dissolved in pure methanol at a stock concentration of 20 mM, stored at −20 °C in the dark and assayed at concentration of 12 µM, which is the half-maximal inhibitory concentration (IC_50_) for simvastatin after 24 h of treatment of MiaPaCa-2 cancer cells in vitro [37]. MiaPaCa-2 cells (ATCC, Manassas, VA, USA) were cultured in Dulbecco’s modified Eagle’s medium (DMEM; Sigma Aldrich, St. Louis, MO, USA) supplemented with 10% fetal bovine serum (heat-inactivated, Thermo Fisher Scientific, Waltham, MA, USA). The cells from two parallel cultures (10 cm^2^ culture dishes) were lysed in the stage of subconfluency using the RLT lysis buffer supplied in a RNeasy Mini Kit (Qiagen, Germantown, MD, USA). Total RNA was isolated by RNeasy Micro Kit (QIAGEN, Germantown, MD, USA) according to the procedure for animal cells. The quantity of RNA was measured by a NanoDrop ND-1000 spectrophotometer (NanoDrop Technologies LLC, Wilmington, DE, USA). The quality of the RNA was analyzed by an Agilent 2100 Bioanalyser (Agilent Technologies, Santa Clara, CA, USA). Those RNA samples that had a RIN (RNA integrity number) above 9 were used for further analysis. Total RNA (150 ng) was amplified using an Illumina TotalPrep RNA Amplification Kit (Ambion, Austin, TX, USA), and 1.5 µg of the amplified RNA was hybridized on the Illumina HumanWG-6_V3 chips (Illumina, San Diego, CA, USA) according to the manufacturer’s protocol. Annotation of detected transcripts was performed using the BioMart package [38] and the Ensembl database (version 47) [39].

Similar to MiaPaCa-2 cells, the transcriptomic profile of human mesenchymal stem cells (AD-MSC; ATCC, Manassas, VA, USA) treated with all existing statins was examined. AD-MSCs in the eighth passage were inoculated into cell culture dishes (⌀60 mm, TPP^®^, Trasadingen, Switzerland) so that the final confluence of the cells ranged between 60–70%. After 24 h of incubation, the cell culture medium (Mesenchymal Stem Cell Basal Medium for Adipose, umbilical, and Bone Marrow-derived MSCs, PCS-500-030, ATCC, Manassas, VA, USA) supplemented with low serum mesenchymal stem cell growth kit for adipose and umbilical-derived MSCs (PCS-500-040; ATCC, Manassas, VA, USA) was discarded and fresh medium containing individual statins at a final concentration of 12 µM, or with the corresponding amount of methanol (99.9%) or clean medium was added. After 24 h of statin treatment, the medium was discarded and 250 µL of RLT RNA Lysis Buffer (part of Rneasy Micro Kit) supplemented with 10 µL of β-mercaptoethanol per 1 mL of RLT was added to each dish. Total RNA was isolated from 0.25 mL of cell lysate using the Rneasy Micro Kit (QIAGEN Sciences Inc, Germantown, MD, USA). The quantity and quality of RNA was determined using a NanoDrop ND-1000 spectrophotometer (NanoDrop Technologies LLC, Wilmington, DE, USA) and an Agilent 2100 Bioanalyzer (Agilent Technologies, Santa Clara, CA, USA). All samples with an RNA Integrity Number (RIN) greater than 7 were subjected to the post-transcriptional microarray analysis using the Clariom S Assay (Applied Biosystems, Waltham, MA, USA). Following the standard protocol, total RNA (250 ng) was amplified and fragmented using the kit and 2300 ng of the resulting fragmented complementary deoxyribonucleic acid (cDNA) was hybridized on the chips according to the manufacturer’s protocol. Each sample group was evaluated in four biological replicates.

The resulting microarray data were processed using the packages oligo [38] and limma [39] of the Bioconductor [40] within the R environment [41]: raw data were summarized, background corrected, quantile normalized and variance stabilized by log_2_ transformation. Moderated *t*-test [42] was used to detect differentially expressed transcripts after fitting the linear model I~Treatment. Storey’s q-value was less than 0.05 [43] and a minimum two-fold change in expression intensity was required to consider differentially transcribed genes. Validation of selected gene expression changes was performed using quantitative polymerase chain reaction (qPCR) [31]. The minimum information about a microarray experiment (MIAME) compliant data was deposited into the ArrayExpress database.

### 2.2. Quantitative Real-Time PCR

Cell cultivation, treatment and lysis, RNA isolation, and its quantification for q-PCR and microarray analyses were performed according to the same protocol. According to the MIQE guidelines [44] several reference genes (4 and 6, respectively) were used. These were selected specifically for each experimental model. The selection was based on the stability of their expression in similar previously published whole-genome transcriptome datasets. Following cross-validation of their stability in our individual PCR experiments, the TBP, RPS9 and GAPDH housekeeping genes were used as the reference for MiaPaCa-2 cells [31], and the RPL13A, HPRT1 and PPIA genes were used as the reference for AD-MSC cells [32]. Reverse transcription was performed using the QuantiTect Reverse Transcription Kit (QIAGEN Inc., Germantown, MD, USA). The qPCR was performed by LightCycler 2.0 system using the LightCycler 480 DNA SYBR Green I Master Kit (Roche Diagnostics, Basil, Switzerland), and the results were analyzed by LightCycler software. The values were further analyzed using the R environment [42] using the ΔΔCp method. The list of amplicons/primers of target and housekeeping genes and the results of the analyses are provided in Supplementary Tables S1 and S2 (*E-MTAB-11579*, *E-MTAB-3979*).

### 2.3. Network Analysis

A functional association network was created based on known and predicted functional interaction partners or selected genes found in microarray analysis that were significantly altered in AD-MSC and MiaPaCa-2 cells after statin treatment at the 12 µM concentration for 24 h. The STRING ([45], https://string-db.org, version 12, accessed on 24 July 2024) networks for *Homo sapiens* were seeded with genes related to apolipoprotein E (*APOE*, *APOC1*, *LRP8*, *LDLR*), angiotensin-converting enzyme 2 (*ACE2*, *AGT*, *MME*), the immune system (*TNFRSF10D*, *TNFRSF6B*, *UNC93B1*, *TNFSF11B*, *MAP3K8*, *IRAK2*, *IL6*, *IL8*) or respiratory failure (*MAP1LC3A*, *SASS6*, *RAB7B*, *CXCL12*, *CXCL16*), and created using the following criteria: physical subnetwork only; highest confidence edges (0.9); text mining, experiment and database sources only. In the first shell, we allowed up to twenty interactors, in the second no more than ten interactors. Heatmaps are provided for all genes within the respective networks that are differentially expressed upon treatment by at least one statin (concentration 12 µmol/L, duration 24 h) in at least one experimental model (MiaPaCa-2, AD-MSC).

## 3. Results

The expression of genes, whose products are reported to be directly associated with complicated COVID-19 disease (*APOE*, *ACE2*, genes encoding proteins involved in innate antiviral immunity and respiratory failure genes) or their interaction partners, was only affected by three of the eight currently existing statins (namely, pitavastatin, cerivastatin and marginally simvastatin) in MiaPaCa-2 cells. In AD-MSC stem cells, the expression of genes of interest was affected by all statins studied, except for pravastatin (Supplementary Figure S2).

### 3.1. Statins and Apolipoprotein E

**Figure 1:**
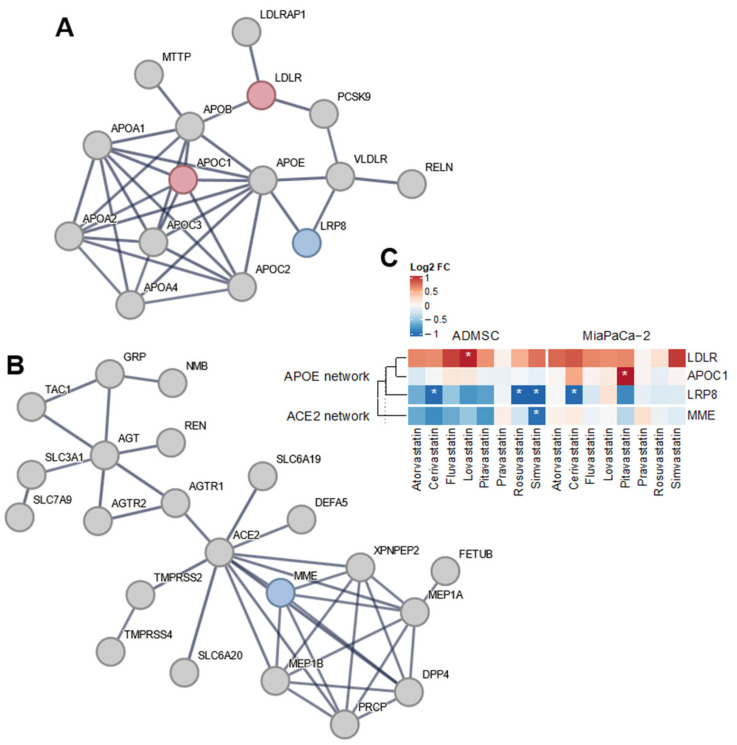
Network analysis of APOE and ACE2 related differentially expressed genes. Among the genes within the APOE network (**A**), *APOC1*, *LDLR*, and *LRP8* genes show differential expression upon treatment by various statins in both experimental models. In contrast, only the *MME* gene is affected by the treatment in the ACE2 network (**B**). Changes in gene expression upon statin treatment are displayed in the heatmap for all differentially expressed genes in the networks (**C**), with statistically significant results denoted by asterisks (|log_2_FC| > 1 and FDR < 0.05). The STRING (https://string-db.org, version 12, accessed on 24 July 2024) networks were seeded with *APOE*, *APOC1*, *LRP8*, and *LDLR* or *ACE2*, *AGT*, and *MME* genes, respectively, in Supplementary Table S3 as described in the Section 2. Genes with overall upregulation (resp. downregulation) upon statin treatment are displayed in red (resp. blue).

*APOE*, encoding apolipoprotein E, which is involved in cholesterol transport, inflammation and other immune responses, is a gene whose variant is the most commonly associated with the complicated COVID-19 disease [29]. The direct expression of the *APOE* gene was not affected by statin treatment (12 µM, 24 h) in any of the cell lines used in this study, but the expression of several genes whose products interact with apolipoprotein E was affected (Figure 1A,C). In MiaPaCa-2 cancer cells, cerivastatin, pitavastatin, and simvastatin increased the expression of the *APOC1* gene (Figure 1A,C), the product of which, together with *APOE* and other apolipoproteins, is an essential component of high-density lipoprotein (HDL), very low-density lipoprotein (VLDL), and chylomicrons. Cerivastatin also decreased the expression of *LRP8* (LDL receptor-related protein 8), the product of which is an LDL receptor, including *APOE*. Simvastatin and rosuvastatin also reduced *LRP8* expression in mesenchymal stem cells, and, in addition, lovastatin increased the expression of a gene encoding the LDL receptor (LDLR, Figure 1A,C).

### 3.2. Statins and Angiotensin-Converting Enzyme 2

The second gene most associated with the progression of COVID-19 is the *ACE2* gene, which encodes the angiotensin-converting enzyme 2 functioning as SARS-CoV-2 receptor [23,24,25]. Statins did not directly alter the expression of *ACE2* in MiaPaCa-2 or AD-MSC cells (Figure 1B,C). However, simvastatin treatment led to a decrease in the expression of the *MME* gene (membrane metalloendopeptidase) in AD-MSC stem cells (log_2_FC = −1.00; *p*-value < 10^−3^). The product of this gene is an enzyme that cleaves, among other substrates, angiotensin 2, which is also the substrate of ACE2 [46].

### 3.3. Statins and Immune System Genes

**Figure 2:**
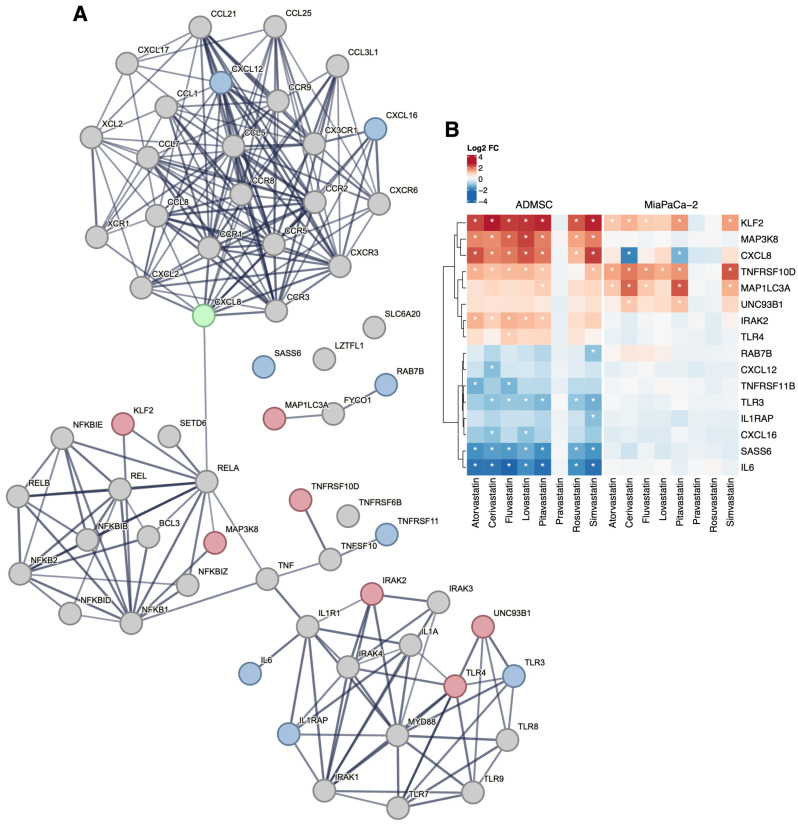
Network analysis of genes related to immune response and respiratory failure. Among the genes within the network (**A**), several members of the chemokine/interleukin family show differential expression upon statin treatment, together with selected TLR related genes and NF-κB interacting genes. The treatment effect is more pronounced in the AD-MSC experimental model. Changes in the gene expression upon statin treatment are displayed in the heatmap for all differentially expressed genes in the networks (**B**), with statistically significant results denoted by asterisks (|log_2_FC| > 1 and FDR < 0.05). The STRING (https://string-db.org, version 12, accessed on 24 July 2024) networks were seeded with the genes listed in Supplementary Tables S4 and S5 as described in the Section 2. Genes with overall upregulation (resp. downregulation/deregulation) upon statin treatment are displayed in red (resp. blue/green). *TNFRSF6B* gene was not detected in the ADMSC cells as the used microarray platform does not target the gene.

Among the genes encoding molecules involved in innate antiviral immunity such as chemokines and their receptors or TLR, which are associated with the complicated course of COVID-19, fluvastatin directly affected the expression of the *TLR4* gene (log_2_FC = 1.00; *p*-value ˂ 10^−9^) in AD-MSC cells (Figure 2). TLR activation affects the production of inflammatory cytokines (tumor necrosis factor-α, TNF-α; and interleukin, IL). In AD-MSC cells, all the statins decreased the expression of *IL6* (Figure 2), the product of which acts as an inflammatory cytokine, playing a crucial role in the terminal differentiation of B cells into Ig immunoglobulin-producing cells. It is also involved in the differentiation of lymphocytes and monocytes [47]. In contrast, all statins increased the expression of the *CXCL8* gene (chemokine (C-X-C motif) ligand 8) encoding IL-8, which activates neutrophils, basophils, and T cells, but not monocytes, as well as the expression of *IRAK2* (Figure 2) encoding interleukin-1 receptor-associated kinase-like 2, which is a kinase that binds to the IL-1 receptor and triggers intracellular signaling cascades leading to upregulation of transcription and stabilization of mRNA. Of the genes encoding TNF-α in stem cells, statins increased the expression of *TNFRSF10D*, and atorvastatin also increased the expression of *TNFRSF11B* (Figure 2) encoding members of the TNF receptor superfamily 10D and 11B, respectively.

Modulation of TLR activity leads to changes in the expression of the gene encoding NF-κB, which may affect the duration of viral persistence and the risk of human-to-human transmission. In AD-MSC stem cells, all statins (except inactive pravastatin) increased the expression of the *MAP3K8* gene that encodes mitogen-activated protein kinase kinase 8. This kinase, an NF-κB interaction partner, is required for the TLR4-mediated activation of the MAPK/ERK pathway in macrophages in response to IL1 in an IRAK-independent manner. This is critical for the production of the pro-inflammatory cytokine TNF-α during immune responses. It is also involved in the regulation of T helper cell differentiation and interferon G expression in T cells. Due to the influence of statins, there was a modulation of TNF family genes (Figure 2); in addition to this, there was also increased regulation of the *UNC93B1* gene in stem cells. The product of this gene, the Unc93 homolog B1 (*C. elegans*), plays an important role in innate and adaptive immunity by regulating nucleotide-sensitive TLRs. It is required for the transport of TLR subunits (including TLR3, TLR7, and TLR9) from the endoplasmic reticulum to endolysosomes, where they can respond to pathogenic nucleotides by activating appropriate signaling pathways, and not least, it plays a role in the elimination of autoreactive B cells.

### 3.4. Statins and Respiratory Failure

We did not observe changes in the expression of any of the six genes clustered in the locus associated with respiratory failure in patients with COVID-19 (*CCR9*, *SLC6A20*, *LZTFL1*, *CXCR6*, *XCR1*, *FYCO1*). However, the expression of some genes encoding the interaction partners of the FYCO1 gene product was affected by the statins’ treatment (Figure 2). Of particular interest are the products of the *RAB7B*, *CXCL12*, and *CXCL16* genes. RAB7B negatively regulates TLR4 signaling in macrophages by promoting lysosomal degradation of TLR4 and promotes megakaryocytic differentiation by increasing NF-κB-dependent IL-6 production. Mutations in the *CXCL12* gene are associated with resistance to human immunodeficiency virus type 1 infection. Related pathways of the *CXCL16* gene product include the CCR5 pathway in macrophages.

*MAP1LC3A* encodes microtubule-associated protein 1A/1B light chain 3A, important markers and effectors of autophagy. SAS-6 is required for centrosome duplication and functions during procentriole formation; SAS-6 ensures that each centriole seeds the formation of a single procentriole per cell cycle.

## 4. Discussion

Five years after the start of the pandemic, the COVID-19 is still one of the most discussed topics, due to its enormous impact on morbidity and mortality. Patients with cardiovascular disease, obesity, high cholesterol and other comorbidities are highly susceptible and at risk for COVID-19. Number of these patients use statins; therefore, some studies have focused on the influence of this medication on the course of COVID-19 in relation to the genetic predisposition of the patients.

Several articles have reported the effect of statins on genes associated with the severe course of COVID-19 disease (as reviewed in [48]). However, the novelty of our study is that it compares the effect of all eight currently existing statins on the expression of genes associated with severe COVID-19, with a particular focus on the organism’s antiviral defense mechanisms. To determine the still unknown molecular mechanisms of multiple organ dysfunction caused by SARS-CoV-2, Zou et al. performed a bioinformatics analysis based on a time-ordered gene co-expression network, with the conclusion that several substances, including statins, are potential target drugs in the treatment of COVID-19 [49]. Statins, without specifying compounds, demonstrated therapeutic effects on lung and tracheal dysfunction by regulating the expression of *RHOA*, *CD40LG* and *CD40* [49]. This is consistent with our previously published data showing a significant effect of cerivastatin, pitavastatin, and simvastatin on *RHOA* gene expression in pancreatic cancer cells cultured in vitro [50]. *RHOA* is activated upon binding of chemokines, cytokines, and growth factors [51,52] and its product is one of the major inducers of lung inflammation and acute lung injury, as it inhibits apoptosis and promotes the proliferation of lung cancer cells [53,54].

A higher concentration of angiotensin 2 (Ang-2) was observed in serum of patients with COVID-19 [55,56,57]. Elevated Ang-2 levels correlated linearly with the severity of lung damage and SARS-CoV-2 dose. Ang-2 induces inflammation (stimulates the IL-6 release), vasculopathy, thrombosis, and coagulopathy [55]. There is an ongoing clinical trial investigating the use of human recombinant soluble ACE2 in patients with severe COVID-19, which neutralizes the viral S protein and reduces organ damage by counterbalancing the inflammatory response [58,59]. A study published in 2013 described a significant increase in *ACE2* gene expression following rosuvastatin administration (5 mg/kg per day) in rats [60]. In contrast to the study concluding that statins minimize the pulmonary dysfunction caused by excess of Ang-2 by directly upregulating of the expression of *ACE2* [61], we found the effect on *ACE2* only for simvastatin, namely by reducing the *MME* gene expression, the product of which cleaves Ang-2 (the substrate for ACE2). Treatment with simvastatin, which reduces *MME* gene expression in stem cells, could lead to increased levels of Ang-2.

ApoE-mediated cholesterol influx has also been found to cause translocation of ACE2 to lipid rafts [62]. In our study, the most effective statins (simvastatin, cerivastatin, and pitavastatin) upregulated *APOC1* in close cooperation with other apolipoproteins, including ApoE [63].

Similarly, we have only observed an indirect effect of statins on NF-κB activity. Its inhibition by statins is thought to limit the “cytokine storm” in patients with severe COVID-19 [64,65]. In our in vitro study, we did not detect direct changes in *NF-κB* gene expression. However, all statins used increased the expression of the NF-κB pathway activator *IRAK-2* in pancreatic cancer cells, and some statins affected the expression of some genes encoding TLRs known to modulate the expression of the gene encoding NF-κB as exemplified by the slight upregulation of *TLR 4* expression by fluvastatin. On the contrary, Chansrichavala et al. observed that atorvastatin at a comparable concentration can reduce *TLR4* expression and thus inhibit the Toll-like receptor-coupled NF-κB pathway in murine pro-BV cells [66]. Inhibition of TLR4 by statins may affect the inflammatory response by reducing levels of pro-inflammatory cytokines through pathways linked to NF-κB. Although in our study only fluvastatin affected the expression of the *TLR4* gene and none of the statins affected NF-κB. In pancreatic cancer cells, all statins significantly downregulated the *IL-6* gene encoding one of the major cytokines associated with COVID-19 [33,67,68]. This is also the reason why COVID-19 is treated with the anti-IL-6 monoclonal antibody tocilizumab [69]. Finally, in clinical trials comparing the effect of tocilizumab with usual care in adults hospitalized with COVID-19, there was no statistically significant benefit on COVID-19 progression [70,71,72] and no reduction in intubation or death [73].

In addition, statins are known inhibitors of MYD88, an adapter protein involved in the Toll-like receptor and IL-1 receptor signaling pathways in the innate immune response [27,74]. A possible stabilization of MYD88 in the presence of external stressors by statins could therefore suggest their role in protecting patients with COVID-19 from the development of an excessive inflammatory response. However, we did not observe a significant impact of statins on the expression of the *MYD88* gene.

Interestingly, we observed an increase in the regulation of the *UNC93B1* gene mediated by pitavastatin in stem cells. Human plasmacytoid predendritic cells (pDC) with a homozygous mutation that causes the lack of functions are unable to differentiate properly after SARS-CoV-2 infection. They also failed to produce IFN-α2, IP-10, and IL-6. It seems possible that the *UNC93B1* product is necessary to recognize viral particles and induce the antiviral response of the immune system [75].

Despite their utility in investigating statin-induced transcriptional changes, the models used do not fully replicate the lung environment or direct viral-host interactions seen in COVID-19, which is the main limitation of our in vitro study. However, we have verified that selected genes under investigation are expressed in our experimental models using the GeneCard database (https://www.genecards.org/, accessed on 24 July 2024), we believe that the models we used are well-suited for the initial exploration of these gene expressions and provide valuable preliminary data and insights. The inclusion of lung cells or immune system cells in future studies could provide more detailed information and further validate our findings. In addition to including lung or immune cells in future experiments to better reflect the in vivo context, our findings open several other perspectives. The observed variability in gene expression responses to individual statins supports the idea of a personalized approach based on patient genotype (e.g., *APOE* variants) and comorbidities. Furthermore, the modulation of immune-related pathways suggests potential relevance of statins in post-COVID-19 conditions, such as long COVID. Their indirect effects on viral entry mechanisms via lipid raft dynamics may also point to a preventive role. As some statins increased the expression of genes linked to immune suppression or viral persistence, future in vivo or clinical studies should consider the timing and dosage of statin administration during the course of infection.

## 5. Conclusions

Statins act on several signaling pathways simultaneously, some of which may support the development of COVID-19, while others may help to suppress it. As the effect of specific statins on the expression of selected genes varies considerably, the impact of statins on disease progression may depend on the type of statin used. Although our experimental models cannot provide definitive conclusions on statin efficacy, our findings offer insights that support further study in more disease-relevant models.

## Data Availability

The datasets generated during and/or analyzed during the current study are available in the ArrayExpress database (repository number E-MTAB-3979 (https://www.ebi.ac.uk/biostudies/arrayexpress/studies/E-MTAB-3979, accessed on 19 May 2022), E-MTAB-11579 (https://www.ebi.ac.uk/biostudies/arrayexpress/studies/E-MTAB-11579, accessed on 19 May 2022)). The list of amplicons/primers of target and housekeeping genes and the results of quantitative real-time PCR are provided in Supplementary Tables S1 and S2. The list of genes used to construct the STRING networks (https://string-db.org, version 12, accessed on 24 July 2024) is given in Supplementary Tables S3 and S5. KEGG pathway hsa05171: Coronavirus disease—COVID-19 is given in Supplementary Figure S1. Gene expression analysis of the members of KEGG pathway hsa05171: Coronavirus disease—COVID-19 is given in Supplementary Figure S2.

## Supplementary Materials

The following supporting information can be downloaded at: https://www.mdpi.com/article/10.3390/biomedicines13071714/s1, Figure S1: KEGG pathway hsa05171: Coronavirus disease—COVID-19 (Kyoto Encyclopedia of Genes and Genomes, https://www.kegg.jp, accessed 24 July 2024); Figure S2: Gene expression analysis of the members of the KEGG pathway hsa05171: Coronavirus disease—COVID-19 (Kyoto Encyclopedia of Genes and Genomes, https://www.kegg.jp, accessed on 24 July 2024). Changes in the gene expression upon statin treatment in both experimental models (AD-MSC and MiaPaCa-2) are displayed in the heatmap for all differentially expressed genes in the pathway. Statistically significant results are denoted by asterisks (|log_2_FC| > 1 and q < 0.05). Table S1: List of primers used for quantitative real-time PCR analyses (AD-MSC cells); Table S2: Quantitative PCR analysis of selected genes (AD-MSC cells) Base 2 logarithm of fold expression changes in statin treated samples vs. controls as detected in RT-qPCR and microarray analyses. The general agreement of the results is clearly visible despite data compression by the microarray method, which is caused by limited dynamic range of the assay. Figures in bold denote statistically significant changes (*p* < 0.1 for RT-qPCR, and FDR < 0.1 for microarray data). The symbols denote n.s. not significant. *p* (resp. FDR) < 0.1, * *p* < 0.05, ** *p* < 0.01, *** *p* < 0.001; Table S3: Effect of statins on the expression of genes encoding *APOE* interaction partners Statistically significant altered transcript expression—|log_2_FC| > 1, q < 0.05, FDR—false discovery rate, experimental model—(a) pancreatic cancer MiaPaCa-2 cells, (b) adipose-derived mesenchymal stem cells AD-MSC, statin concentration—12 µmol/L, duration of action—24 h; Table S4: Effect of statins on the expression of genes encoding Toll-like receptors and genes encoding their interaction partners. Statistically significant altered transcript expression—|log_2_FC| > 1, q < 0.05, FDR—false discovery rate, experimental model—(a) pancreatic cancer MiaPaCa-2 cells, (b) adipose-derived mesenchymal stem cells AD-MSC, statin concentration—12 µmol/L, duration of action—24 h; Table S5: Other loci associated with COVID-19-induced respiratory failure and significantly affected by statins. Statistically significant altered transcript expression—|log_2_FC| > 1, q < 0.05, experimental model—(a) pancreatic cancer MiaPaCa-2 cells, (b) adipose-derived mesenchymal stem cells AD-MSC—12 µmol/L, duration of action—24 h.

## Abbreviations

The following abbreviations are used in this manuscript:

ACE2	Angiotensin-converting enzyme 2
AD-MSC	Adipose tissue-derived human mesenchymal stem cells
APOE	Apolipoprotein E
ARDS	Acute respiratory distress syndrome
CCR9	C-C chemokine receptor type 9
COVID-19	Coronavirus disease 2019
CXCR6	C-X-C motif chemokine receptor 6
CXCL12	Chemokine (C-X-C motif) ligand 12
CXCL16	Chemokine (C-X-C motif) ligand 16
ERK	Extracellular signal-regulated kinases
FC	Fold change
FYCO1	FYVE and coiled-coil domain-containing protein 1
GO	Gene ontology
GSEA	Gene set enrichment analysis
HDL	High-density lipoprotein
HMG-CoA reductase	3-hydroxy-3-methylglutaryl-coenzyme A reductase
IL	Interleukin
IRAK2	Interleukin-1 receptor-associated kinase-like 2
KEGG	Kyoto encyclopedia of genes and genomes
LDL	Low-density lipoprotein
LDL-R	Low-density lipoprotein receptor
LZTFL1	Leucine zipper transcription factor-like protein 1
MAP1LC3A	Microtubule-associated protein 1 light chain 3α
MAP3K8	Mitogen-activated protein kinase kinase kinase 8
MIAME	Minimum information about a microarray experiment
MiaPaCa-2	Human cells from pancreatic carcinoma
MME	Membrane metalloendopeptidase
NF-κB	Nuclear factor kappa-light-chain-enhancer of activated B cells
RAB7B	Ras-related protein Rab-7b
RIN	RNA integrity number
SASS6	Spindle assembly 6 homolog (*C. elegans*)
SLC6A20	Sodium-dependent imino acid transporter 1
TLR	Toll-like receptors
TNF	Tumor necrosis factor
TNFRSF10D	Tumor necrosis factor receptor superfamily member 10D
TNFRSF11B	Tumor necrosis factor receptor superfamily member 11B
UNC93B1	Unc93 (*C. elegans*) homolog B1
VLDL	Very low-density lipoprotein
XCR1	Chemokine XC receptor 1
