# Supplementary material for: In Vitro Investigation of Statin Effects on Genes Associated with Severe COVID-19 in Cancerous and Non-Cancerous Cells
Source: Biomedicines. 2025 Jul 14;13(7):1714. doi: 10.3390/biomedicines13071714 (PMC12292488; doi:10.3390/biomedicines13071714)
Supplement: Supplementary file 1 [file biomedicines-13-01714-s001.zip › Supplementary Material Figure S2.pdf]

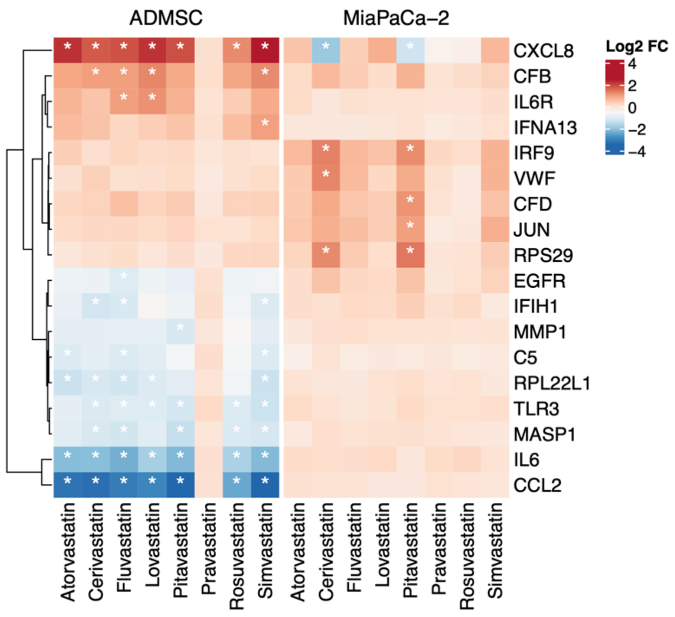

Supplementary Figure S2. Gene expression analysis of the members of the KEGG pathway hsa05171: *Coronavirus disease - COVID-19* (Kyoto Encyclopedia of Genes and Genomes, https://www.kegg.jp, accessed on July 24, 2024). Changes in the gene expression upon statin treatment in both experimental models (AD-MSC and MiaPaCa-2) are displayed in the heatmap for all differentially expressed genes in the pathway. Statistically significant results are denoted by asterisks ($|\log_2 FC| > 1$ and $q < 0.05$).
