# Supplementary material for: In Vitro Investigation of Statin Effects on Genes Associated with Severe COVID-19 in Cancerous and Non-Cancerous Cells
Source: Biomedicines. 2025 Jul 14;13(7):1714. doi: 10.3390/biomedicines13071714 (PMC12292488; doi:10.3390/biomedicines13071714)
Supplement: Supplementary file 1 [file biomedicines-13-01714-s001.zip › Supplementary Material Tables S1_S2_S3_S4_S5.pdf]

Supplementary Table S1. List of primers used for quantitative real-time PCR analyses (AD-MSC cells)

Gene	Type	Left primer	Right primer
**TNFRSF10D**	*Target*	AGTTCCTGTACCACGACCAG	CAGGATGGTGGTCACTGTCT
**IL8**	*Target*	AGACAGCAGAGCACACAAGC	AGGAAGGCTGCCAAGAGAG
**IL6**	*Target*	AGACAGCCACTCACCTCTTC	TTTCACCAGGCAAGTCTCCT
**LRP8**	*Target*	CAGTCACTGCCGCTGTTATC	GACATGGCCAATCTGAGCAG
**SASS6**	*Target*	TCGCAGGCTGTTTGAAATGT	ATTGAACCTGTGCCTGCAAG
**Rpl13**	*Reference*	CCGCTCCAAACTCATCCTCT	GCCATACGGAGACTAGCGAA
**HPRT1**	*Reference*	GACCAGTCAACAGGGGACAT	GTGTCAATTATATCTTCCACAATCAAG
**PPIA**	*Reference*	CCTAAAGCATACGGGTCCTG	TTTCACTTTGCCAAACACCA
**TBP**	*Reference*	GAACATCATGGATCAGAACAACA	ATAGGGATTCCGGGAGTCAT

Supplementary Table S2. Quantitative PCR analysis of selected genes (AD-MSC cells)

Gene	Method	Cerivastatin	Pitavastatin	Simvastatin	Fluvastatin	Atorvastatin	Lovastatin	Rosuvastatin	Pravastatin
**TNFRSF10D**	*RT-qPCR*	**2.94****	**2.36****	**2.3****	**2.58***	**2.26***	2.74 n.s.	1.17 n.s.	0.43 n.s.
	Microarray	**1.16*****	**1.08*****	**1.18*****	**1.25*****	**1.35*****	**1.27*****	**0.29***	0.03 n.s.
**IL8**	*RT-qPCR*	**3.36***	**3.82***	**3.93***	**3.45***	**3.86***	**3.32***	2.66 n.s.	0.77 n.s.
	Microarray	**1.79*****	**1.92*****	**2.46*****	**1.90*****	**2.26*****	**2.24*****	**1.16*****	0.10 n.s.
**IL6**	*RT-qPCR*	**-1.62***	**-3.19****	**-2.12****	-2.24 n.s.	-2.39 n.s.	**-1.51***	**-1.42***	-0.26 n.s.
	Microarray	**-2.11*****	**-2.19*****	**-2.17*****	**-2.33*****	**-2.13*****	**-1.78*****	**-1.74*****	0.10 n.s.
**LRP8**	*RT-qPCR*	-1.75 n.s.	-0.49 n.s.	-0.59 n.s.	-0.34 n.s.	-1.62 n.s.	-0.58 n.s.	-0.87 n.s.	0.13 n.s.
	Microarray	**-1.03*****	**-0.73*****	**-1.07*****	-0.52n.s.	**-0.61*****	**-0.78*****	**-1.07*****	-0.03 n.s.
**SASS6**	*RT-qPCR*	**-2.15****	-0.75 n.s.	-1.97 n.s.	-1.01 n.s.	-3.49 n.s.	-1.44 n.s.	**-3.69***	-0.05 n.s.
	Microarray	**-1.55*****	**-1.75*****	**-1.66*****	**-1.60*****	**-1.72*****	**-1.38*****	**-1.41*****	0.16 n.s.

Base 2 logarithm of fold expression changes in statin treated samples vs. controls as detected in RT-qPCR and microarray analyses. The general agreement of the results is clearly visible despite data compression by the microarray method, which is caused by limited dynamic range of the assay. Figures in bold denote statistically significant changes ($p < 0.1$ for RT-qPCR, and FDR < 0.1 for microarray data). The symbols denote n.s. not significant. p (resp. FDR) < 0.1, * $p < 0.05$, ** $p < 0.01$, *** $p < 0.001$.

Supplementary Table S3. Effect of statins on the expression of genes encoding APOE interaction partners.

a)

Statin	*APOC1*		*LRP8*	
	log_2FC	FDR	log_2FC	FDR
Simvastatin	1.11	$< 10^{-3}$	-	-
Cerivastatin	1.58	$< 10^{-3}$	-1.10	$< 10^{-5}$
Pitavastatin	1.68	$< 10^{-6}$	-	-

b)

Statin	*LDLR*		*LRP8*	
	log_2FC	FDR	log_2FC	FDR
Simvastatin	-	-	-1.31	$< 10^{-5}$
Cerivastatin	-	-	-1.03	$< 10^{-3}$
Lovastatin	1.09	$< 10^{-4}$	-	-
Rosuvastatin	-	-	-1.07	$< 10^{-3}$

Statistically significant altered transcript expression - $|log_2FC| > 1$, $q < 0.05$, FDR – false discovery rate, experimental model - a) pancreatic cancer MiaPaCa-2cells, b) adipose-derived mesenchymal stem cells AD-MSC, statin concentration - 12 μmol/L, duration of action - 24 h.

Supplementary Table S4. Effect of statins on the expression of genes encoding Toll-like receptors and genes encoding their interaction partners.

a)

**Statin**	***TNFRSF10D***		***TNFSF11B***		***MAP3K8***		***IRAK2***		***IL6***		***IL8***	
	log_2FC	*p*	log_2FC	*p*	log_2FC	*p*	log_2FC	*p*	log_2FC	*p*	log_2FC	*p*
Simvastatin	1.18	$< 10^{-10}$	-	-	1.80	$< 10^{-12}$	-	-	-2.17	$< 10^{-14}$	2.46	$< 10^{-17}$
Cerivastatin	1.16	$< 10^{-10}$	-	-	1.00	$< 10^{-11}$	1.00	$< 10^{-5}$	-2.11	$< 10^{-13}$	1.79	$< 10^{-13}$
Pitavastatin	1.80	$< 10^{-9}$	-	-	1.85	$< 10^{-12}$	1.10	$< 10^{-6}$	-2.19	$< 10^{-13}$	1.92	$< 10^{-14}$
Rosuvastatin	-	-	-	-	1.49	$< 10^{-9}$	-	-	-1.74	$< 10^{-11}$	1.26	$< 10^{-9}$
Lovastatin	1.27	$< 10^{-11}$	-	-	2.38	$< 10^{-15}$	1.25	$< 10^{-7}$	-1.78	$< 10^{-11}$	2.24	$< 10^{-15}$
Fluvastatin	1.25	$< 10^{-11}$	-	-	2.80	$< 10^{-13}$	1.33	$< 10^{-8}$	-2.33	$< 10^{-14}$	1.90	$< 10^{-14}$
Atorvastatin	1.35	$< 10^{-12}$	-1.26	$< 10^{-4}$	1.78	$< 10^{-11}$	1.34	$< 10^{-8}$	-2.13	$< 10^{-13}$	2.26	$< 10^{-15}$

b)

**Statin**	***TNFRSF10D***		***TNFRSF6B***		***UNC93B1***	
	log_2FC	FDR	log_2FC	FDR	log_2FC	FDR
Simvastatin	1.55	$< 10^{-3}$	-	-	-	-
Cerivastatin	-	-	-1.15	$< 10^{-3}$	-	-
Pitavastatin	1.81	$< 10^{-4}$	-	-	1.22	$< 10^{-8}$

Statistically significant altered transcript expression - $|log_2FC| > 1$, $q < 0.05$, FDR – false discovery rate, experimental model - a) pancreatic cancer MiaPaCa-2 cells, b) adipose-derived mesenchymal stem cells AD-MSC, statin concentration - 12 µmol/L, duration of action - 24 h.

Supplementary Table S5. Other loci associated with COVID-19-induced respiratory failure and
significantly affected by statins.

a)

**Statin**	***MAP1LC3A***	
	log_2FC	FDR
Simvastatin	1.52	$< 10^{-4}$
Cerivastatin	1.73	$< 10^{-3}$
Pitavastatin	1.52	$< 10^{-4}$

b)

**Statin**	***MAP1LC3A***		***SAS-6***		***RAB7B***		***CXCL12***		***CXCL16***	
	log_2FC	*p*	log_2FC	*p*	log_2FC	*p*	log_2FC	*p*	log_2FC	*p*
Simvastatin	-	-	-1.66	$< 10^{-8}$	-1.08	$< 10^{-6}$	-	-	-	-
Cerivastatin	-	-	-1.55	$< 10^{-7}$	-	-	-1.15	$< 10^{-8}$	-1.47	$< 10^{-4}$
Pitavastatin	1.00	$< 10^{-5}$	-1.75	$< 10^{-8}$	-	-	-	-	-	-
Rosuvastatin	-	-	-1.41	$< 10^{-6}$	-	-	-	-	-	-
Lovastatin	-	-	-1.38	$< 10^{-6}$	-	-	-	-	-1.09	$< 10^{-3}$
Fluvastatin	-	-	-1.60	$< 10^{-7}$	-	-	-	-	-	-
Atorvastatin	-	-	-1.72	$< 10^{-8}$	-	-	-	-	-	-

Statistically significant altered transcript expression - $|log_2FC| > 1$, $q < 0.05$, experimental model - a) pancreatic cancer MiaPaCa-2 cells, b) adipose-derived mesenchymal stem cells AD-MSC, statin concentration - 12 µmol/L, duration of action - 24 h.
