# Supplementary figures and images for: In Vitro Investigation of Statin Effects on Genes Associated with Severe COVID-19 in Cancerous and Non-Cancerous Cells
Source: Biomedicines. 2025 Jul 14;13(7):1714. doi: 10.3390/biomedicines13071714 (PMC12292488; doi:10.3390/biomedicines13071714)

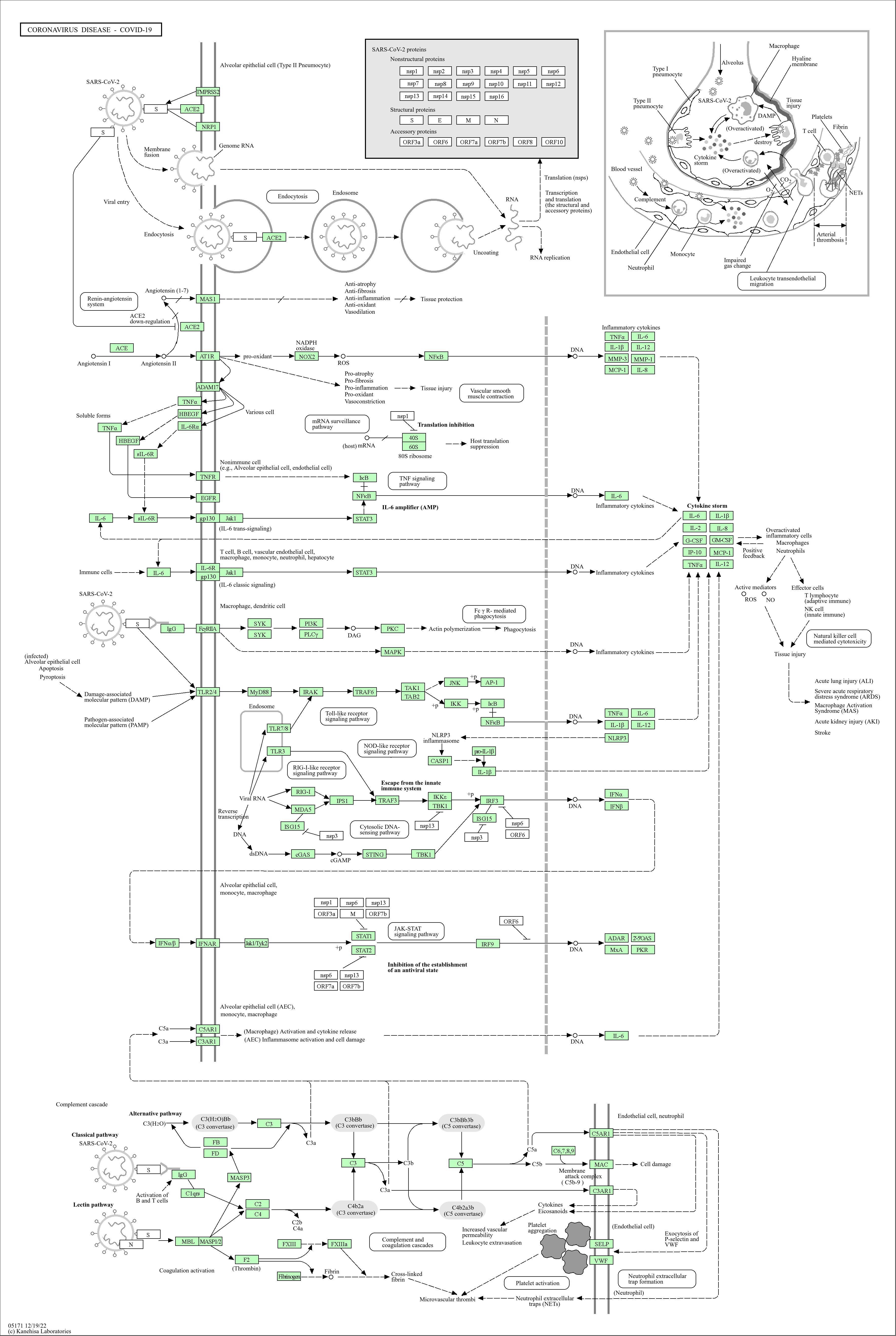

Supplement: Supplementary file 1 [file biomedicines-13-01714-s001.zip › Supplementatary Material_Figure_S1.png]
